# Putative photosensitivity in internal light organs (organs of Pesta) of deep-sea sergestid shrimps

**DOI:** 10.1038/s41598-023-43327-z

**Published:** 2023-09-26

**Authors:** Tamara Frank, Jamie Sickles, Danielle DeLeo, Patricia Blackwelder, Heather Bracken-Grissom

**Affiliations:** 1 Halmos College of Arts and Sciences, Nova Southeastern University, Dania Beach, FL 33004 USA; 2 Department of Invertebrate Zoology, National Museum of Natural History, Smithsonian Institution, Washington, DC 20013 USA; 3 Institute of Environment and Department of Biology, Florida International University, North Miami, FL 33181 USA

**Keywords:** Genetics, Physiology, Ocean sciences

## Abstract

Many marine species can regulate the intensity of bioluminescence from their ventral photophores in order to counterilluminate, a camouflage technique whereby animals closely match the intensity of the downwelling illumination blocked by their bodies, thereby hiding their silhouettes. Recent studies on autogenic cuticular photophores in deep-sea shrimps indicate that the photophores themselves are light sensitive. Here, our results suggest photosensitivity in a second type of autogenic photophore, the internal organs of Pesta, found in deep-sea sergestid shrimps. Experiments were conducted onboard ship on live specimens, exposing the animals to bright light, which resulted in ultrastructural changes that matched those seen in crustacean eyes during the photoreceptor membrane turnover, a process that is crucial for the proper functioning of photosensitive components. In addition, RNA-seq studies demonstrated the expression of visual opsins and phototransduction genes in photophore tissue that are known to play a role in light detection, and electrophysiological measurements indicated that the light organs are responding to light received by the eyes. The long sought after mechanism of counterillumination remains unknown, but evidence of photosensitivity in photophores may indicate a dual functionality of light detection and emission.

## Introduction

More than 80% of known bioluminescent species are marine, but emission wavelengths and types, i.e. a spew, a slime, a simple light organ, or a complex light organ(s), vary amongst species^1^. Blue light transmits the furthest through seawater, as the longer wavelengths are absorbed and the shorter wavelengths are scattered and absorbed^2,3^ and therefore most marine bioluminescence is blue^1^. Additionally, downwelling light from surface waters is intense enough to stimulate visual responses from animals living at the bottom of the mesopelagic zone (200–1000 m), and thus most mesopelagic organisms possess a single, blue sensitive visual pigment to maximize sensitivity to the available light^4^. The large eyes and sophisticated vision of some predators can detect differences in contrast such as the body of an organism blocking downwelling light and thereby revealing a silhouette against a dimly lit background^1^. Therefore, many species in the mesopelagic zone, particularly those that perform vertical migrations, have developed a strategy called counterillumination, where they use their photophores to produce bioluminescent emissions that replicate the wavelength, intensity, and angular distribution of ambient light being blocked by the body, to camouflage their silhouette and avoid detection^1,5^.

**Figure 1:**
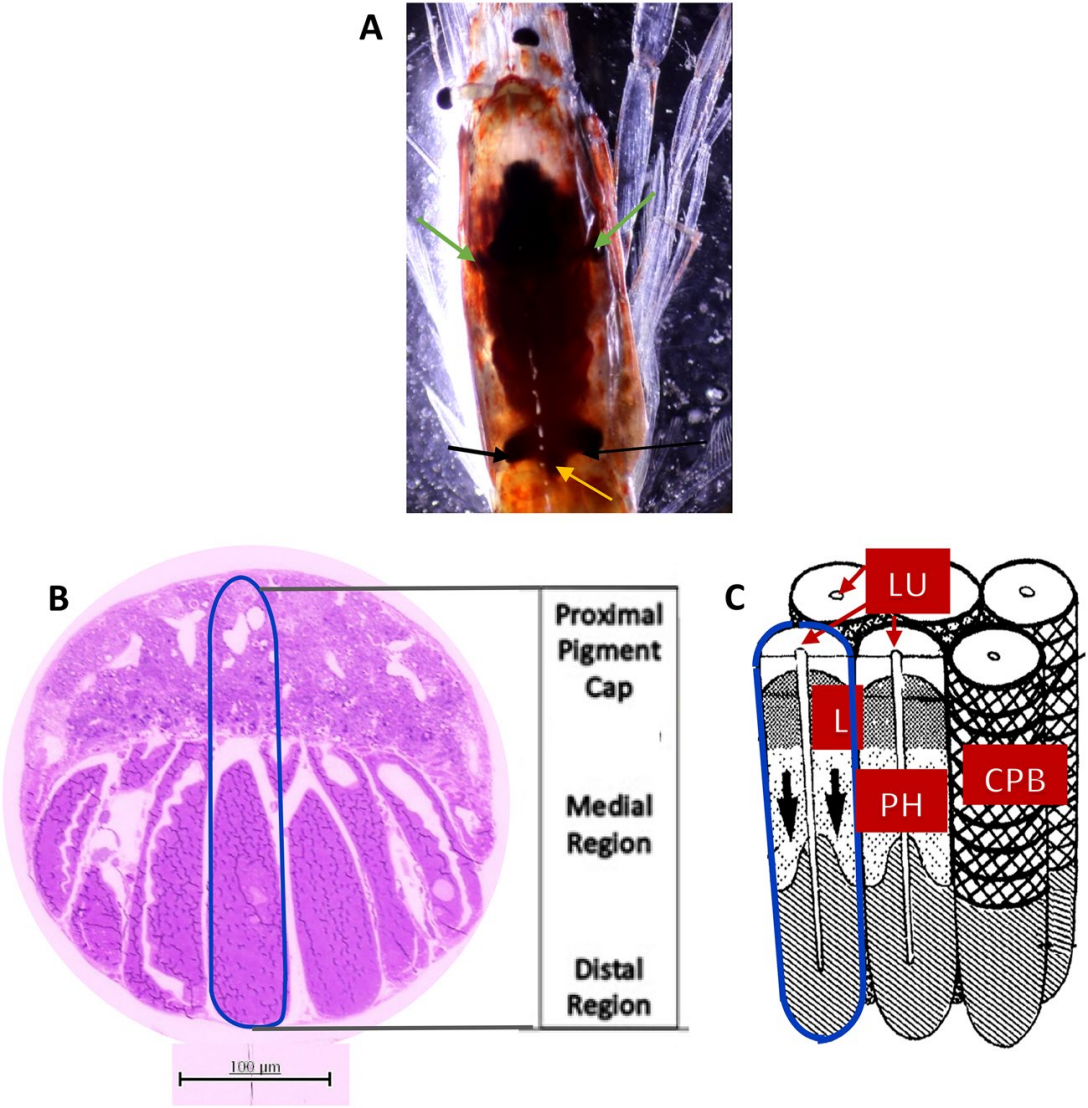
General structure of internal photophores in a *Allosergestes sargassi.* (**A**) Dorsal view of organ of Pesta includes anterolateral (green arrow) and posterolateral organ pairs (black arrows), and a medial organ (not found in all *Sergestes)* in between the postlateral organ pairs (yellow arrow). (**B**) Light microscope image of a dissected organ of Pesta. The proximal pigment cap is a prominent structure that caps each individual tubule (outlined in blue) and creates a parabolic shape over the entire organ. Individual tubules are sectioned into three regions: proximal, medial, and distal. (**C**) Schematic drawing from Denton et al. (1985) of translucent tubule structure in hepatic photophores. The diffuse lipid layer (L) separates proximal and medial tubule regions, while the carotenoid pigment border (CPB) creates a sheath throughout these regions. The medial region is presumed to be the photogenic region (PH) that generates bioluminescent light. Bioluminescent emissions are directed downward (arrows) and are filtered in the distal region. The central lumen (LU) extends throughout all three tubule regions (proximal, medial, and distal).

The counterillumination mechanism uses ventral, lateral, or internal light organs known as photophores to produce a bioluminescent emission that closely matches the intensity of the surrounding ambient light. Photophores produce bioluminescence through chemical reactions always involving the oxidation of a luciferin, the light-emitting molecule, catalyzed by a luciferase enzyme, and sometimes a photoprotein (catalyzing protein bound together with a luciferin and oxygen)^1^. Photophores are of two types, bacteriogenic and autogenic. Bacteriogenic photophores house symbiotic bioluminescent bacteria, while autogenic photophores are equipped with the necessary substrates and enzymes to produce bioluminescence^6^. Many species of deep-sea shrimps of the Sergestidae family possess autogenic photophores that are either dermal (lensed or unlensed), consisting of external photophores that appear continuous with the chitin, or internal organs of Pesta that are continuous with, but distinct from, gastrointestinal organs within the cephalothorax (Fig. 1A)^7–9^. Both types of photophores contain light-producing cells, optical structures, and associated components (pigments, lipids)^8,10–15^^)^ but differ vastly in ultrastructure.

Organs of Pesta, also known as internal or hepatic photophores, are found only in what was previously known as the genus “*Sergestes*”^16^ (now reclassified into seven genera) and have a species specific arrangement always including anterior and posterior organs and, in some species, medial organs (Fig. 1A)^17,18^. Each organ is composed of several individual translucent tubules divided into proximal, medial and distal regions (Fig. 1B). These tubules are lined by a single layer of columnar epithelial cells containing mitochondria, endoplasmic reticulum (ER), Golgi, nuclei, lipids, and microvilli, and have external microtubule channels^11^. The distal portion of epithelial cells consists of a microvilli brush border that forms a central lumen throughout the tubule regions (proximal, medial, and distal)^8,11,15^. The proximal region is composed of carotenoid pigments and lipids and caps translucent tubules, thus directing bioluminescent light downward. The distal portion of the proximal region has a concentration of lipids that acts as a diffuse layer^11^. The medial region is suspected to be the photogenic region where the bioluminescence is produced, due to the presence of paracrystalline platelets, presumed to be the photogenic cells^11,19,20^. Finally, the distal region serves to filter bioluminescent light emissions, and therefore few organelles are found in the distal tips of organs of Pesta^11^. While a considerable portion of the body of the sergestids with organs of Pesta is transparent (e.g., Fig. 8), the hepatopancreas and foregut are not, and the organs of Pesta bracket these opaque regions. Latz^15^ has also determined that as the size of the opaque regions increase with body length, so does the size of the light organs, indicating that these light organs are optimized for light emission to hide these opaque regions. Laboratory experiments have induced counterillumination in live specimens of *Eusergestes similis*^5,21,22^, but the long sought-after mechanism for how these animals can so closely match the irradiance of light blocked by their bodies remains unknown. One type of autogenic photophore (cuticular) in two species of deep-sea oplophorid shrimps, *Janicella spinicauda* and *Systellaspis debilis* was found to contain visual opsins and phototransduction genes, components required for light detection in most animals^23,24^ and common to ocular photoreceptors (eyes). Light exposure experiments further demonstrated changes in photophore organelles consistent with those seen in crustacean photoreceptors during photoreceptor membrane turnover, in which degraded or photodamaged photosensitive structures are synthesized during dark adaptation^23^. The cellular components in photoreceptors that play a role in membrane turnover, that are also found in photophores, include mitochondria, microvilli, lysosomes, nuclei, ER and Golgi bodies^10,11^. If these photophores are indeed sensitive to light, then light exposure should elicit changes in the number or morphology of these organelles similar to those documented in photoreceptors. Here, we utilized several techniques to determine whether light affected these organelles in the organs of Pesta in a manner consistent with light sensitivity, if the molecular machinery required for photoperception was present, and whether the organs themselves respond to light.

## Results

A variety of techniques were used in this study to determine if the organs of Pesta in sergestid shrimps contain light sensitive tissue. Live specimens were collected without exposure to light on several cruises. We used transmission electron microscopy to determine if controlled exposure to various durations and irradiances of light produced changes in organelles present in the organs of Pesta similar to changes seen in the same organelles in photoreceptors. RNA-sequencing was used to determine if the organs of Pesta contained visual opsins and phototransduction genes, which can play a role in light detection in photoreceptors. Lastly, electrophysiological techniques were used to determine if the organs themselves could produce an electrical signal in response to a light stimulus.

### Ultrastructural results

Changes in light exposed tissues included the formation of cytoplasmic organelles such as pinocytotic vesicles, multivesicular bodies, multilamellar bodies, amorphous bodies, dense bodies—henceforth referred to as “lipids”—, membrane whorls in endoplasmic reticulum, fragmented Golgi bodies, and contact sites between “lipids” and organelles. These cellular morphological changes were the same in both *Allosergestes sargassi* and *Parasergestes armatus*, and therefore data from these species were combined for this analysis. The formation of these cytoplasmic organelles and organelle-to-“lipid” contact sites were observed in medial and distal tubule regions in light exposed tissues, but were not found in proximal regions, and therefore only medial and distal regions are included in this analysis. The structures referred to as “lipids” would require biochemical testing to determine their true nature, which was not carried out in the current study, but their ultrastructural characteristics (morphology, size, and inclusion at organelle contact sites) are analogous to lipids identified as such in current biological lipid research^25–28^.

**Figure 2:**
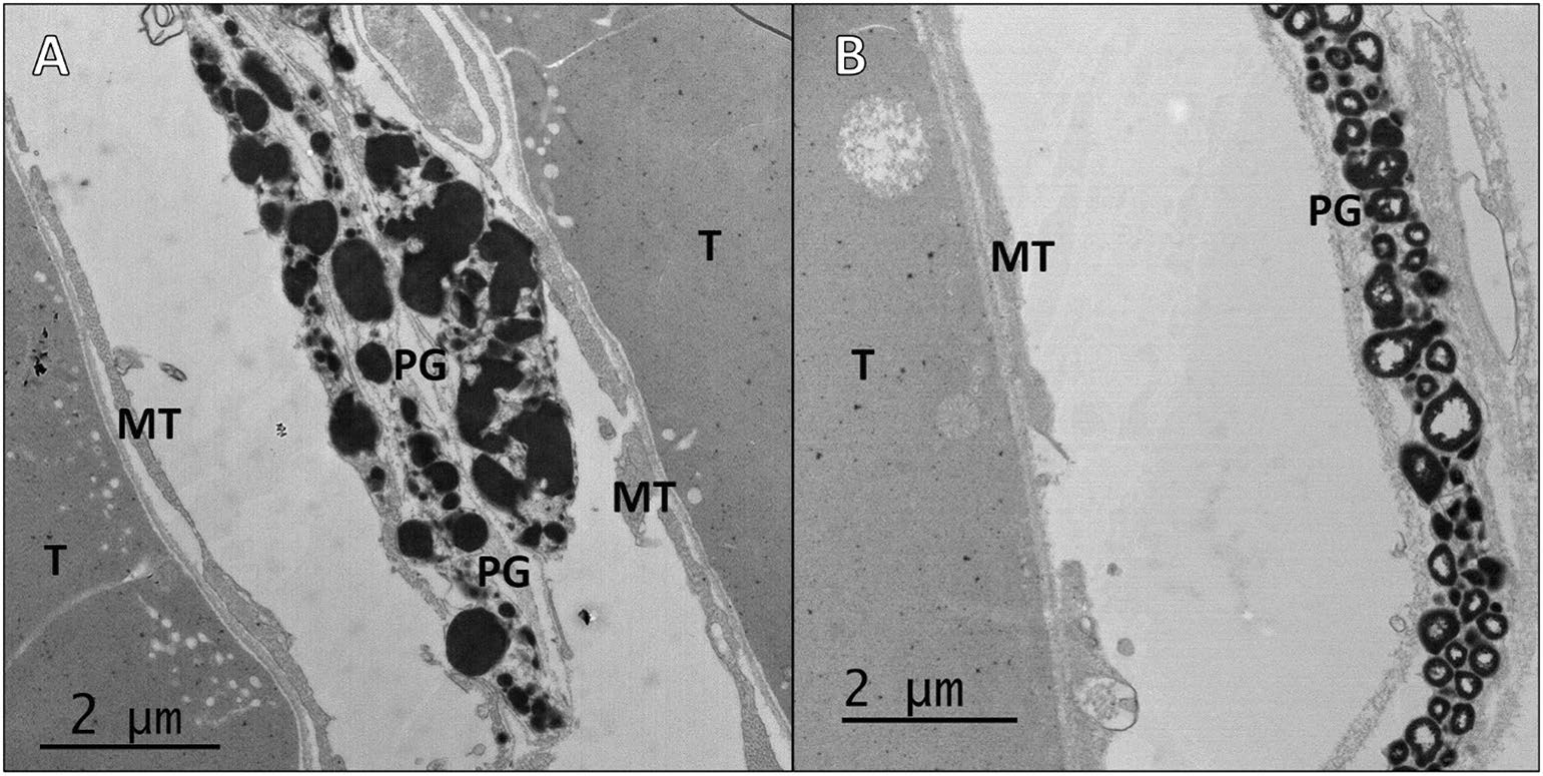
Pigment granules (PG) border exterior lateral sides of tubules (T) and are adjacent to microtubules (MT). (**A**) Pigment granules in control tissues displayed normal morphologies. (**B**) Granules in light exposed tissues were sometimes seen with empty centers.

**Figure 3:**
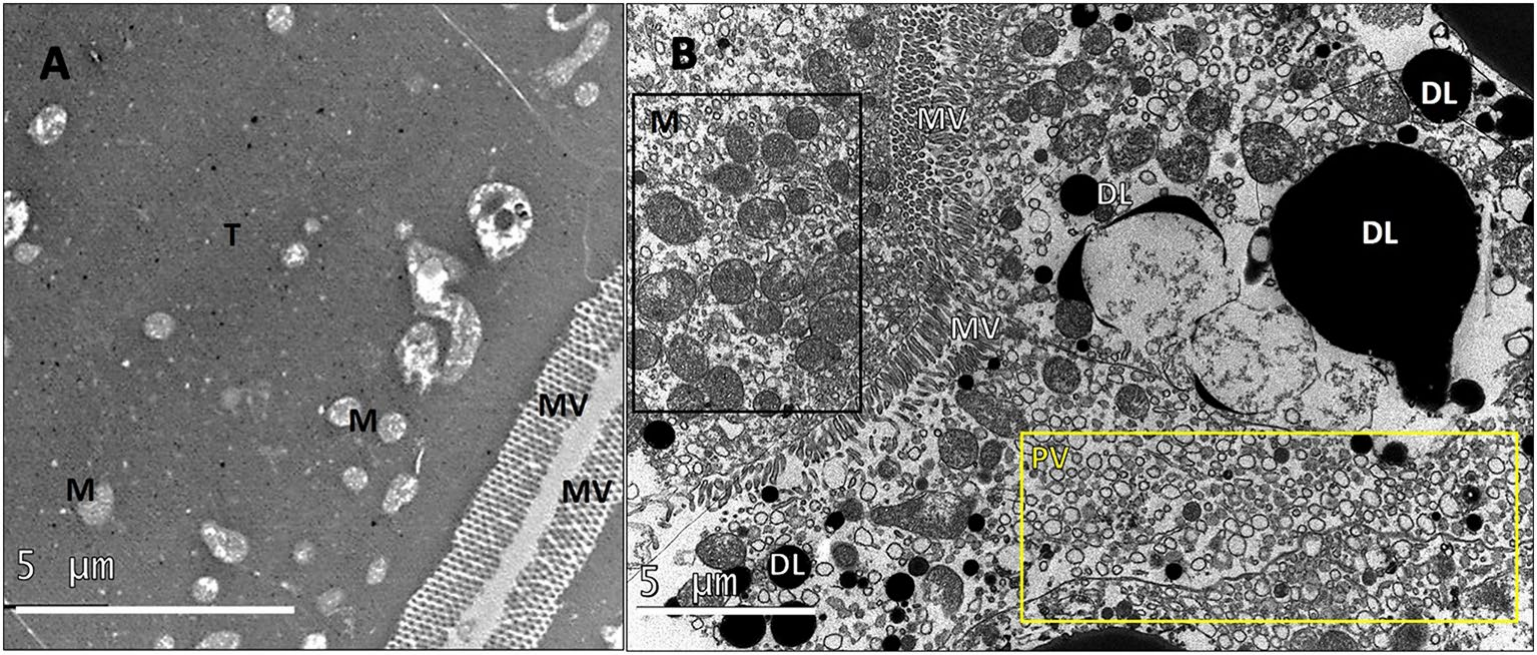
Tissue in the medial tubule region. (**A**) Tubule (T) tissue in the controls contained mitochondria (M) and displayed dense cytoplasm adjacent to well organized microvilli (MV) brush borders. (**B**) In light exposed tissues pinocytotic vesicles (PV, yellow box) pinched off from microvilli (MV) at tubule brush borders and entered the surrounding cytoplasm. These sites had an abundance of mitochondria (M, black box) and dense “lipids” (DL).

**Figure 4:**
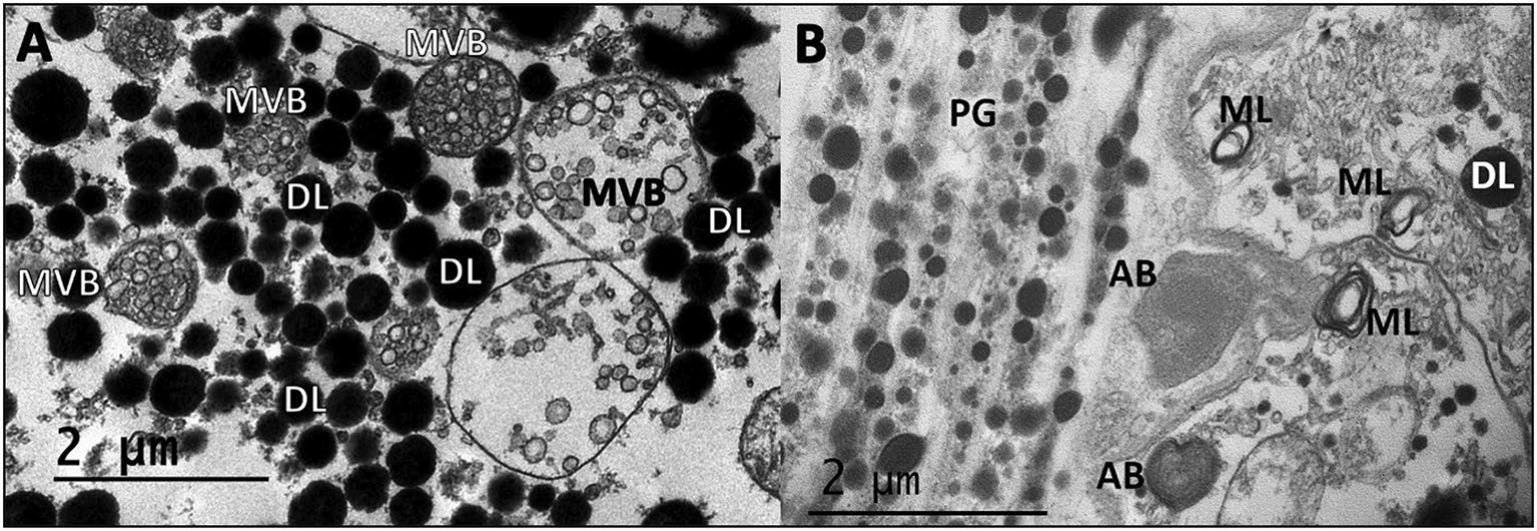
Cytoplasmic organelles formed in the tubule cytoplasm in the medial region of light exposed tissue. (**A**) Multivesicular bodies (MVB) composed of pinocytotic vesicles in tubule cytoplasm were surrounded by dense “lipids” (DL). Pinocytotic vesicles in smaller MVBs were more compact than larger MVBs. (**B**) Small multilamellar structures (ML) in tubule cytoplasm were adjacent to pigment granules (PG) and associated with amorphous bodies (AB) and DLs.

In both control and experimental tissues, strands of carotenoid pigments between tubules extended along their exterior lateral sides, throughout the medial region, and were adjacent to microtubules. In light exposed tissues, pigment granules were sometimes seen with empty centers (Fig. 2B), a morphology that was not seen in control tissues (Fig. 2A). Pinocytotic vesicles, multivesicular bodies, multilamellar bodies, and amorphous bodies were only seen in light exposed tissues. Medial tubule regions in the controls displayed dense tubule cytoplasm that contained mitochondria organelles and well-organized microvilli brush borders (Fig. 3A) but in light exposed tissues, pinocytotic vesicles were seen pinching off microvilli brush borders into the surrounding cytoplasm and were associated with mitochondria and dense “lipids” (Fig. 3B). Pinocytotic vesicles were internalized into multivesicular bodies (MVB) that ranged in size between 0.2 and 3 µm (Fig. 4A). MVBs were common near brush border sites but could be found throughout tubule cytoplasm. Small (0.5 µm) multilamellar bodies (MLBs) could be found throughout tubule cytoplasm as well, in both medial and distal regions, and were associated with amorphous and dense “lipids” in medial regions (Fig. 4B). Large (10 µm) MLBs were only seen in the central lumen of light exposed medial tissues, between a myriad of paracrystalline bodies and “lipids”.

**Figure 5:**
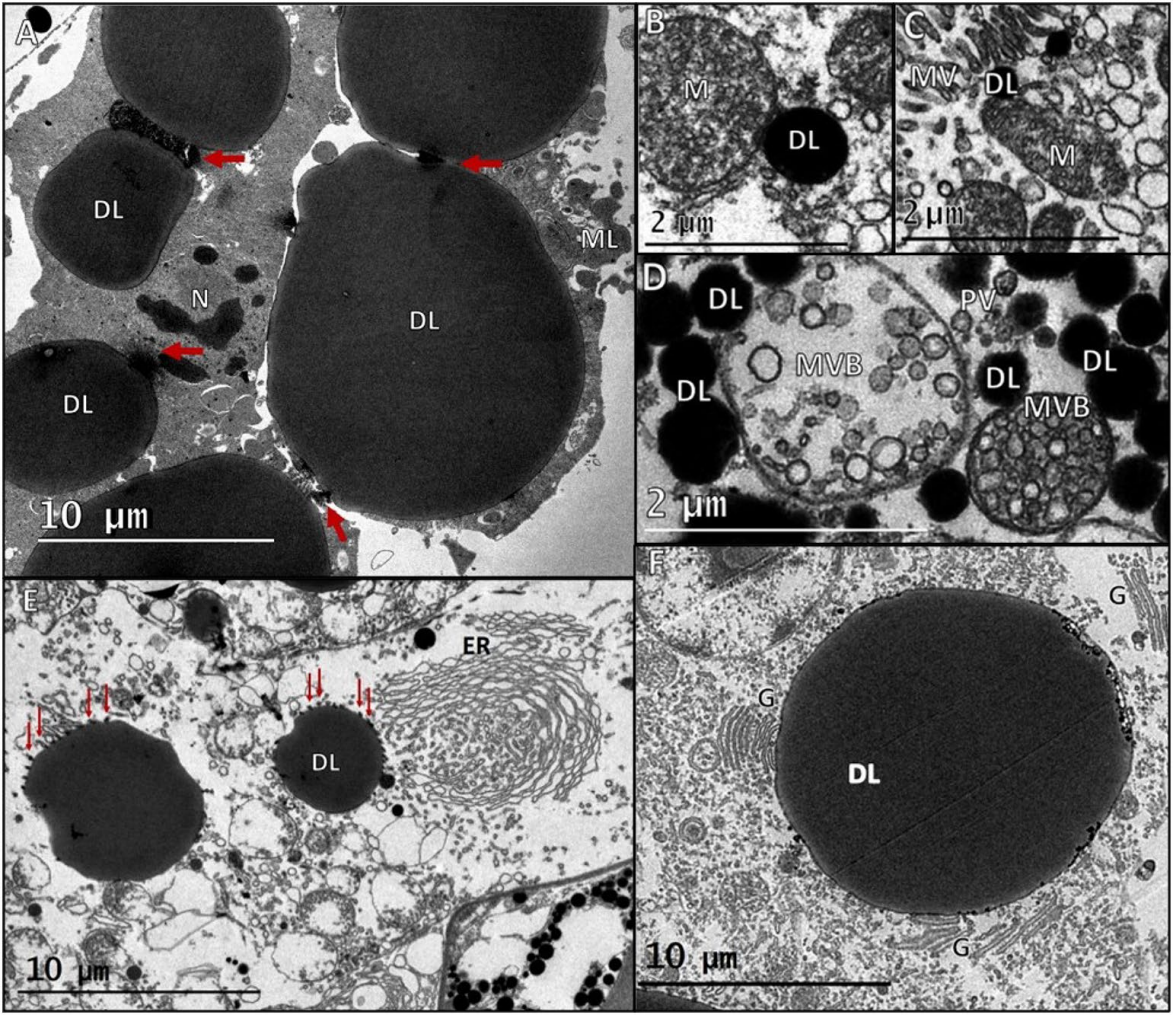
Dense “lipids” are in direct contact with several organelles in medial and distal tubule regions in light exposed tissues. (**A**) Dense “lipids” (DL) in the distal region appear to be sharing electron dense material (red arrows) with other DLs. A DL in the bottom left of the image appears to be sharing similar electron dense material with a tubule nucleus (N). A small multilamellar structure (ML) is also in contact with a DL. **(B**) A DL in the medial region is in direct contact with a mitochondrion (M). (**C**) A DL in the medial region is in direct contact with microvilli (MV) and a mitochondrion (M). (**D**) DLs in the medial region in direct contact with multivesicular bodies (MVB) and nearby pinocytotic vesicles (PV). (**E**) Vesicles (red arrows) formed on DL exteriors when structures were in contact with the endoplasmic reticulum (ER), which displayed multiple membrane whorls. (**F**) Several Golgi (**G**) organelles were simultaneously in contact with a single, large DL.

**Figure 6:**
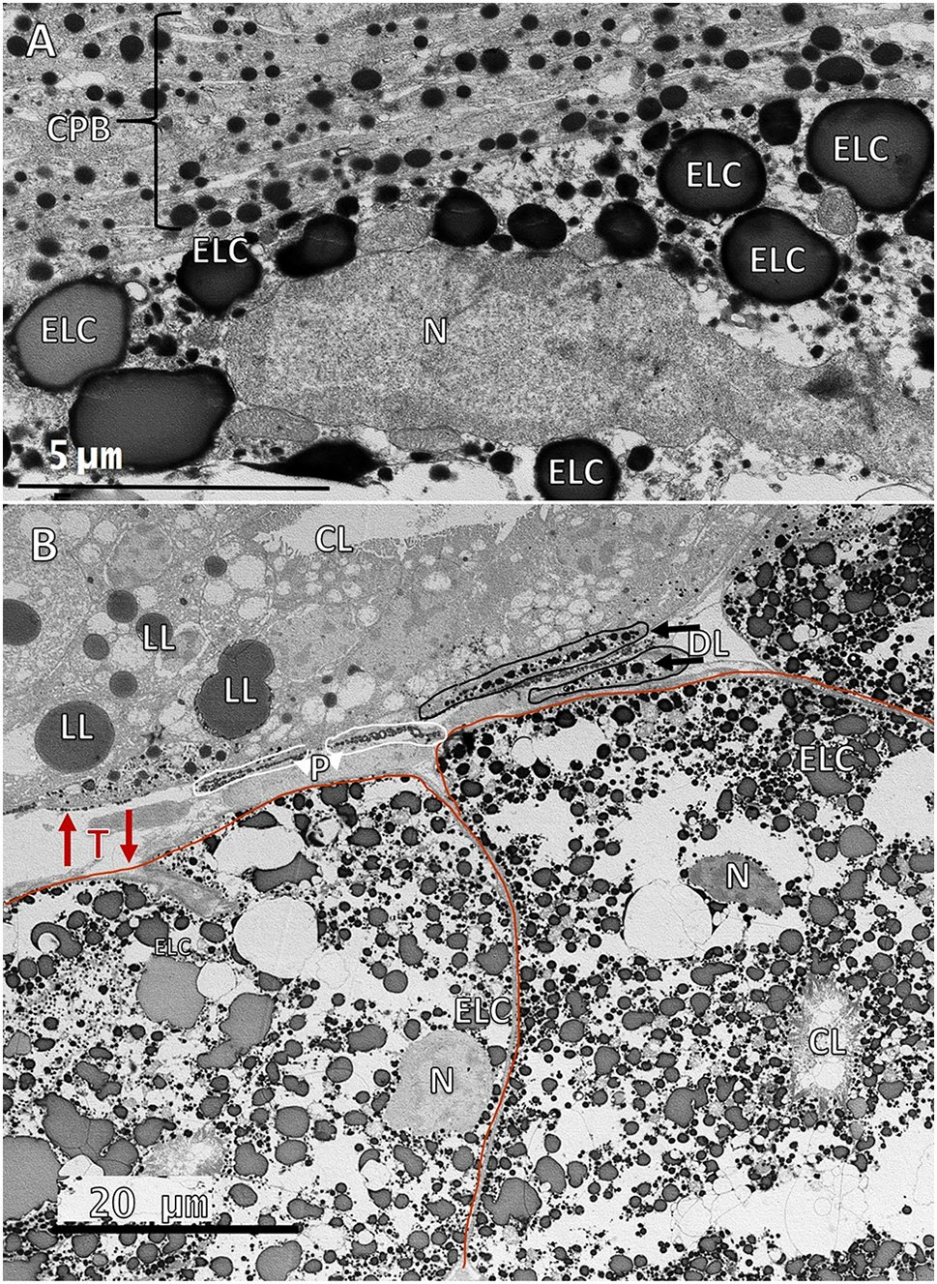
Distribution of different lipid types. (**A**) “Lipids” with an electron dense “phospholipid” layer and an electron lucent core (ELC) in the medial region of control tissue, surround a tubule nucleus (N) that is adjacent to the carotenoid pigment border (CPB). (**B**) A portion of four distal region tubules from a light exposed photophore where all three “lipid” types are present: dense “lipids” (DLs), “lipids” with an electron dense “phospholipid” layer and an electron lucent core (ELC), and electron lucent “lipids” (LLs). ELCs are seen surrounding tubule nuclei (N) and appear to replace dense tubule (T) cytoplasm in the two tubules outlined in red. Pigment granules (P, white arrowheads) and dense lipids (DL, black arrows) enclosed in membranes are seen between the top and bottom tubules. Central lumen (CL), nucleus (N).

In both control and light exposed tissues, “lipids” were the most common structures throughout the tubules. “Lipid" diameters were measured in control and light exposed tissues and analyzed between medial and distal tubule regions. Based on morphological differences, it appears that there are three different types of “lipids” present in organs of Pesta (see Fig. 6B): (1) electron dense “lipids” (DLs), (2) “lipids” with an electron dense “phospholipid” layer and an electron lucent core (ELCs), and (3) electron lucent “lipids” (LLs). DLs were the most ubiquitous and diversely shaped, being either circular (Fig. 5D), oblong or amoeboid (Fig. 5A). In medial tissue regions, the mean diameter of 473 DLs from nine bright light exposed photophores were significantly larger than the mean diameter of 426 DLs from four control photophores (0.77 ± 0.085 vs. 0.549 µm ± 0.015; Mann–Whitney Wilcoxon, *p* ˂ 0.001). In distal tissue regions, the mean diameter of 503 DLs from nine bright light exposed photophores was also significantly greater than the mean diameter of 531 DLs from four control photophores (1.174 µm ± 0.082 vs. 0.63 µm ± 0.012; Mann–Whitney Wilcoxon, *p* ˂ 0.001). The largest measured DL diameter (15 µm) was found in light exposed distal regions surrounded by other large DLs that all appeared to be sharing electron dense material (Fig. 5A). In tissues exposed to light, DLs were often in contact with each other and were the only structures to come in contact with several organelles including microvilli, mitochondria, tubule nuclei, endoplasmic reticulum, and Golgi bodies.

In light exposed tissues, DLs commonly interacted with mitochondria and microvilli at pinocytotic vesicle formation sites (Fig. 5B,C), as well as MVBs in the cytoplasm (Fig. 5D). Endoplasmic reticulum (ER) and Golgi bodies were numerous, large, and well-defined in light exposed tissues, but were not seen in the controls. DLs in contact with the ER formed small vesicles on their exterior surface and the ER displayed membrane whorls (Fig. 5E). Often, several Golgi bodies made simultaneous contact with a single, large (13–15 µm) DL, and some Golgi appeared to be fragmented (Fig. 5F). In both control and light exposed tissues, DLs were also seen in contact with the nuclear envelope and within the nucleoplasm of tubule nuclei, typically displaying an undulating membrane.

In both control and light exposed tissues, DLs and ELCs were found throughout translucent tubules or within the central lumen, displaying a mostly amoeboid and fluid morphology, and could also be seen surrounding tubule nuclei regardless of light exposure (Fig. 6A,B). Additionally, ELCs often appeared to replace dense tubule tissue in the distal region of light exposed tissues (Fig. 6B). In medial tissue regions, the mean diameter of 219 ELCs from nine bright light exposed tissues was significantly greater than the mean diameter of 488 ELCs from four control photophores ((3.616 µm ± 0.122 vs. 2.707 µm ± 0.063; T-test, p ˂ 0.001). However, in distal tissue regions, the mean diameter of 322 ELCs from nine bright light exposed tissues was not significantly different than the mean diameter of 393 ELCs from four control photophores ((1.776 µm ± 0.110 vs. 1.372 µm ± 0.040; Mann–Whitney Wilcoxon, p = 0.3163). Although not significantly different, ELC diameters in distal regions were consistently smaller than 5 µm in the controls but could measure up to 15 µm in light exposed tissues.

The largest measured “lipid” diameters were found in the LLs, which were exclusively located in distal tubule regions (Fig. 6B). The mean diameter of 132 LLs from nine bright light exposed tissues was significantly greater than the mean diameter of 209 LLs from four control photophores (5.88 µm ± 0.438 vs. 0.83 µm ± 0.020; Mann–Whitney Wilcoxon, p ˂ 0.001). LL diameters in the controls were less than or equal to 1.7 µm and nearly half of LL diameters in light exposures were greater than 5 µm, with 25 µm being the largest diameter measured.

### RNA-sequencing results

An average of 33.9 M paired-end reads were generated per organ of Pesta sample (n = 4) with a mean quality score of 37.6. These data are available on the NCBI’s Sequence Read Archive (SRA) database under Bioproject: PRJNA690607. The tissue-specific de novo transcriptome assembly for the organs of Pesta of *Parasergestes armatus* contained 136,507 contigs with a mean length of approximately 761 base pairs (bp) and a contig N50 of 1200 bases. Among those contigs, 88.6% of universal single-copy arthropod orthologs were identified (Complete: 80.5% [Single: 8.6%, Duplicated: 71.9%], Fragmented: 8.1%, Missing: 11.4%, n: 1066), indicating a fairly complete, tissue-specific assembly. These BUSCO scores are comparable to the tissue-specific transcriptomes assembled de novo from the cuticular photophores of deep-sea oplophorid shrimps^23,24^.

**Figure 7:**
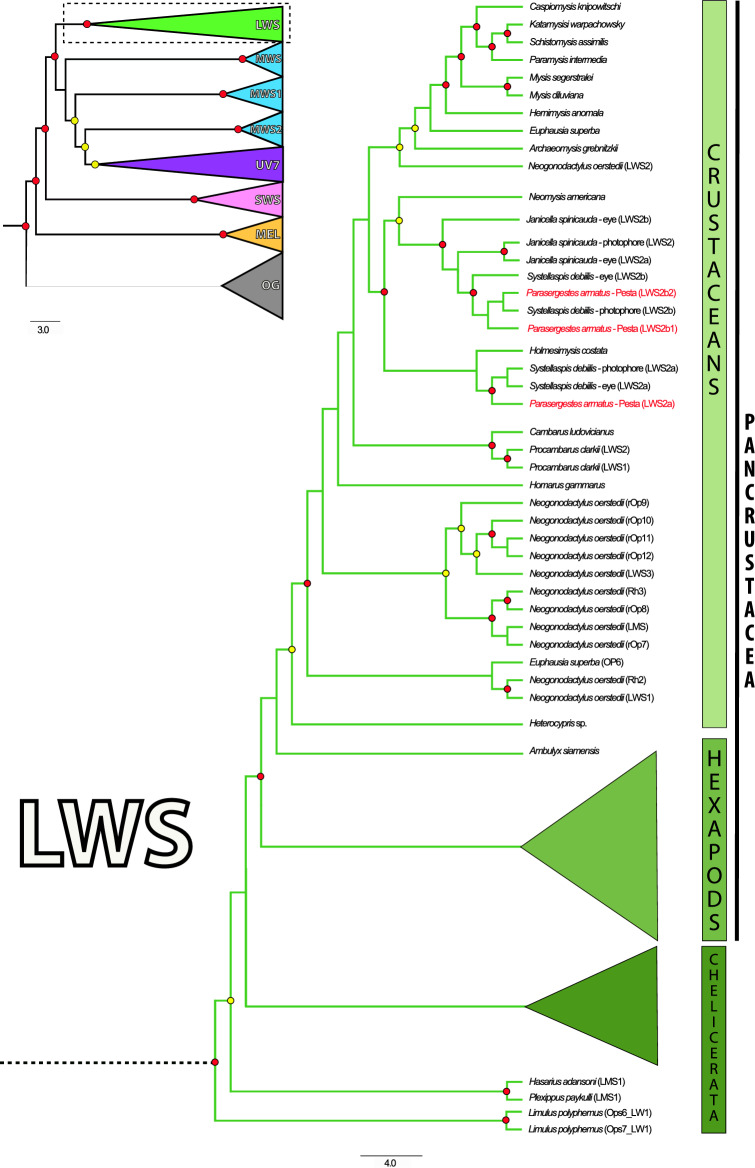
Targeted arthropod opsin phylogenetic tree. (Upper left) Opsin tree reconstruction comprising 283 visual rhabdomeric (r-opsins) and closely related melanopsins (see DeLeo and Bracken-Grissom 2020 for more sequence details) from targeted arthropods. The r-opsins curated from the *Parasergestes armatus* organs of Pesta transcriptome were aligned with a reference opsin dataset comprising visual opsins across a range of measured spectral sensitivities as well as non-visual opsins and related G-protein coupled receptors (outgroups). The spectral sensitivities of the r-opsins clades (*SWS* short wavelength sensitive, *MWS* mid-wavelength, *LWS* long wavelength, *UV7* RH7 unknown) were inferred from these reference datasets. Organs of Pesta opsins (labeled red) correspond to a well-supported LWS clade (enlarged). These opsins more specifically fall under a crustacean specific-grouping of the LWS2 clade, that comprises a majority of the other putative LW opsins. The second well-supported LWS clade is specific to chelicerates (LWS1). Significant triplicate bootstrap support is indicated by red circles (SH- aLRT > 80, aBayes > 0.95 and UFBoot > 95) and significant duplicate bootstrap support is indicated by yellow circles (SH-aLRT > 80 or UFBoot > 95, and aBayes > 0.95).

Phylogenetically-informed annotation (PIA) analyses of the organs of Pesta transcriptome from *P. armatus* revealed three visual r-opsins belonging to a single long-wavelength sensitive clade (LWS2) (Fig. 7). The opsins (designated LWS2a, LWS2b1 and LWS2b2) were identical (LWS2a, 100% amino acid similarity) or near-identical (LWS2b1 and b2, 99% amino acid similarity) to the LWS2 opsins recovered by DeLeo and Bracken-Grissom^24^ from the cuticular photophores (LWS2a and b, respectively) of the deep-sea oplophorid *Systellaspis debilis* though there were some differences at the nucleotide level. Relative to each other, the LWS2a and LWS2b (1 and 2) opsins were ~ 80% similar at the amino acid level. However, the third putative opsin recovered (LWS2b2) from the organs of Pesta is a probable sequence isoform of the LWS2b1 r-opsin considering it is 98% similar and the node separating LWS2b1 and LWS2b2 lacks significant bootstrap support (Fig. 7). In addition to the r-opsins, phototransduction pathway analyses of the organs of Pesta assembly identified a majority of the major pathway components including pathway regulating Gq-proteins, and the cascade terminators—retinal degeneration (rdg) and arrestin (Arr) (genes included Arr, DAGK, GPRK2, Gqα, Gqβ, R-opsin, PKC, rdgC). The absence of the calcium ion (Ca^2+^) channel-transient receptor potential (trp) from the organs of Pesta transcriptome may be due to the lack of expression at the time of sampling or fundamental differences in phototransduction signaling pathways, which have been noted in other metazoans^29^, and are currently unknown for deep-sea shrimps.

### Electrophysiological results

**Figure 8:**
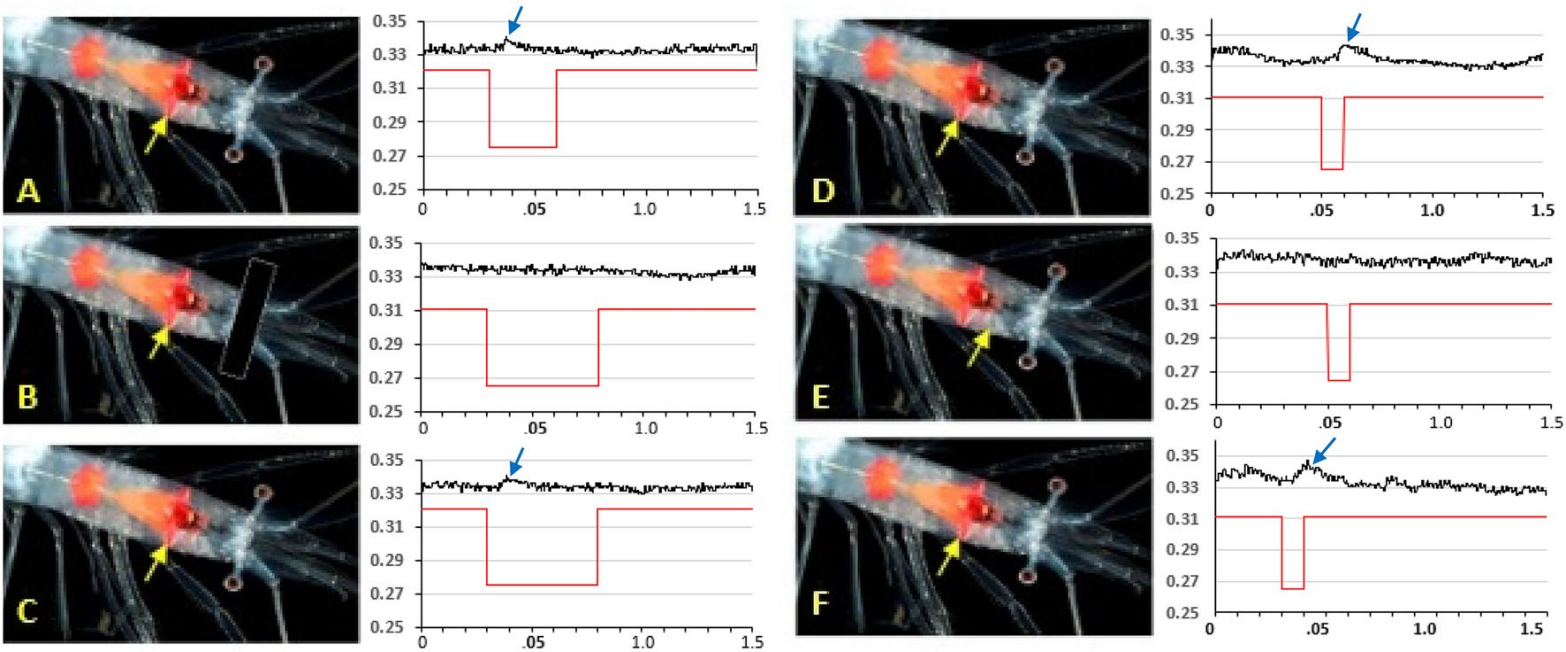
Electrophysiological responses of organs of Pesta, in animals with intact eyes. Images of photophore structure are of *Eusergestes arcticus* (©David Shale, used with permission), which has an identical photophore structure to *Deosergestes seminudus*, the species from which responses were recorded. Yellow arrows indicate the position of the electrode in the anterolateral organ. The X-axis is mV; the y axis is seconds. Blue arrows indicate small responses (black traces) recorded from the organ. Red traces indicate the duration of the light stimulus. (**A**–**C**) were in the same specimen; (**D**–**F**) are from another individual. (**A**) Response recorded from anterior organ. (**B**) No response from the organ when eyes were shielded. (**C**) Return of response when shield was removed. (**D**) Response recorded from another specimen from the anterior organ. (**E**) No response when the electrode was placed in the muscle tissue. (**F**) Return of the response when the electrode was replaced in the organ.

Initial experiments (five) were conducted on live specimens with the eyes removed, and no responses were recorded from any organs of Pesta. Serendipitously, an animal was accidentally set up for experimentation without removing the eyes and based on the resulting responses from the organ of Pesta, all subsequent experiments were conducted on animals with intact eyes. When the stimulus light was not shielded from the eyes (so that both the eyes and the organ of Pesta could see the light), a small response was recorded by the electrode placed in the anterolateral portion of organ of Pesta (Fig. 8A). When the eyes were shielded from the light, no response was recorded from the same electrode (Fig. 8B). Once the light shield was removed, a small response was again visible (Fig. 8C). These data were replicated in six different animals. In another set of experiments with eyes unshielded, the electrode was again placed in the anterolateral portion of the light organ, and after verifying the presence of a small response (Fig. 8D), the electrode was removed under dim red light and placed in muscle tissue just anterior to the light organ. After allowing 30 min in complete darkness, no response could be recorded from the muscle tissue (Fig. 8E). The electrode was then removed from the muscle tissue under dim red light and replaced in the anterolateral portion of the light organ, and after 30 min of dark adaptation, a small response was again visible (Fig. 8F), verifying that the response was coming from the organ of Pesta and was not the “echo” of the response from the eye conducted through the seawater bath. These responses, or lack of responses in muscle tissue, were replicated in an five additional animals. The responses ranged from 10 to 15 µV, and that, together with the response latency, made them distinguishable from the background noise level of 3–4 µV. The duration of the stimulus had no effect on the recorded response. The lack of a response when the eyes were shielded, as well as when the electrode was in the muscle tissue, served as controls from which background noise could be determined.

## Discussion

Prior to this study, only one previous ultrastructural study on the organs of Pesta^11^ was performed, which provided a general overview of the organs as described in the introduction. However, there is no description in the methods of whether the specimens were collected alive or what light levels they may have been exposed to during collections and fixations, so comparisons with our results would not be valid. As Herring’s^11^ description of the general structure is the same as what was found in the current study, this discussion will focus on the effects of light exposure on the ultrastructure of these organs.

Crustacean photoreceptor membrane turnover is a naturally occurring process that is in sync with an animal’s circadian rhythm that occurs in the following well-known sequential phases: (1) screening pigment migration^41^, (2) pinocytotic vesicle formation via microvilli, Golgi, and ER^34^, (3) secondary endocytosis of pinocytotic vesicles into multivesicular bodies (MVBs), (4) the accumulation of acid phosphatase in the late stages of MVBs to form secondary lysosomes, such as combination bodies (CBs), and multilamellar bodies (MLBs), (5) the compression of these MLBS to form amorphous and dense bodies and (6) the formation of lipids^30–40,42,44–53^. This process naturally occurs on a circadian cycle, but high-intensity light, i.e. light levels that exceed those in an animal’s natural habitat, dramatically increases the formation of cytoplasmic organelles and disruption in the morphology photosensitive organelles^48^.

Exposing organs of Pesta to bright light resulted in the presence of cytoplasmic organelles identical to those formed during the light-adapted phase of photoreceptor membrane turnover in crustacean compound eyes, first described in the spiny lobster by Lowe^31^ and subsequently described in a number of crustacean species^30,32^. A substantial number of pinocytotic vesicles were found adjacent to microvilli brush borders (that line the central tubules [Fig. 1C]) of light exposed tissues. In crustacean retinas, exhausted photosensitive membranes that make up microvilli break down into pinocytotic vesicles, enter the retinular cytoplasm, and are taken up by multivesicular bodies (MVBs) via secondary endocytosis^33,34^. Photosensitive membranes continue to break down in MVBs with the help of degrative enzymes, possibly derived from the endoplasmic reticulum and Golgi apparatus, which promote further breakdown and formation of multilamellar bodies (MLBs)^33–37^. The presence of pinocytotic vesicles, MVBs, and MLBs in light exposed organs of Pesta, suggests a photosensitive ability in these photophores, perhaps within the microvilli brush borders where the vesicle formation was most apparent.

Two types of multilamellar structures were present in organs of Pesta: (1) small MLBs (0.5–2 µm) with less than four lamellar inclusions that were found in both control and light exposed tissue and (2) large MLBs (5–10 µm) with many lamellar inclusions that were exclusively located in light exposed tissues, between the diffuse lipid layer and paracrystalline platelets in the central lumen of medial tubule regions. MLBs function to further breakdown photosensitive membranes in light-adapted crustacean retinas using enzymes^34^, but other evidence suggests that MLBs may store photosensitive components to aid in photopigment synthesis, i.e., ommochromes used in photoregeneration of metarhodopsin back to rhodopsin^37,38^ and energy storage during dark-adaptation^38^ which may explain the presence of MLBs in control tissues. Due to their possible role in photopigment synthesis in photoreceptors, this may also be the location of opsin expression in photophores. In the late stages of degradative membrane turnover in photoreceptors, additional enzymes in MLBs accumulate, condense, and undergo a series of electron dense reactions to form amorphous and dense bodies^35,36,39,40^. It has been hypothesized that these “dense bodies” are lipids or lipofuscin granules^37^ but this has not been verified. The placement of large MLBs in the central lumen may indicate that these structures play a unique or important role in the counterillumination process as they were surrounded by microvilli and between dense populations of “lipids” on one side and paracrystalline platelets (the supposed photogenic cells) on the other.

“Lipids” were the most common and diverse structures found throughout tubule regions in both control and light-exposed tissues. Light exposure resulted in significantly larger “lipid” diameters (with the exception of DLs and ELCs in distal regions), an increase in the number and size of endoplasmic reticulum (ER) and Golgi bodies, and widespread “lipid” to organelle contact. “Lipid” diversity would allow for a greater refractive index of light^54^ which may be enhanced by increasing “lipid” diameters during the onset of light^55^. Dense “lipids” came into contact with several organelles including microvilli, mitochondria, nuclei, ER, and Golgi bodies in light exposed tissues. Phospholipids make up a portion of microvilli membranes in crustacean rhabdoms^33,53,56^ and of lipids in general. The phospholipid monolayer in lipids contains enzymes and up to 160 different types of proteins^57^ that influence lipid to organelle contact^58^ suggesting that lipids may also influence cellular processes in organs of Pesta.

ER and Golgi organelles were not prominent in the control tissue but were abundant in light exposed tissues. Golgi and ER typically multiply during the onset of light in photoreceptors due to elevated levels of cellular activity and energy requirements^43–45,48,59^ to synthesize membranes, lipids, visual pigments, and proteins^36,39,42,53^. These organelles were only found in proximity to, or in direct contact with, dense “lipids”. The ER is the main site of lipid body formation in plants, animals, and microorganisms due to the enzymes and proteins produced in ER systems^60^ so lipids remain in contact with the ER throughout the lipid life cycle for the purpose of lipid and protein trafficking, and to respond to ER stress and ER-associated degradation^26^. Cells depend on ER and Golgi stability for vesicular trafficking, a process vital for cellular communication and function^27^. However, cellular deformities, such as the whorled or fragmented membranes (ER and Golgi, respectively) seen in some of the light exposed photophores, indicate unfavorable cellular conditions^48^ which can interrupt vesicular trafficking and initiate a non-vesicular trafficking method^25,28^. Non-vesicular trafficking is where lipids are used instead of ER or Golgi to maintain cellular functions and exchange material^25,26,28,61^.

Dense “lipids” (DLs) and “lipids” with an electron dense “phospholipid” layer and an electron lucent core (ELCs) were seen in contact with nuclear envelopes, but DLs could also be found within the nucleoplasm. This may be a normal occurrence since there are shared proteins between lipids and nuclei for nuclear-droplet communication and chromatin remodeling (reviewed in^26^). Lipid to organelle associations and/or contact sites may be physiologically important for cellular activity, such as homeostasis, membrane biosynthesis, metabolism, and protein regulation, as recently suggested in several proteomic reviews in cellular biology^25–28,61^. Current biological research indicates that lipids are crucial components for cellular activity^27^, and therefore should be further investigated in autogenic photophores to better develop our understanding of the mechanisms of counter illumination. While DL, ELC and LL diameters were significantly larger in tubule regions in tissues exposed to light, no conclusions can be drawn about these results without confirming the identity of proteins in the “lipid” membrane or of amino acids contained in the hydrophobic core. Although most of the evidence pertaining to the effects of light on lipids in visual systems remains inconclusive, the interaction of “lipids” (if eventually identified as such) with several organelles in organs of Pesta suggests that lipids play an important role in the function of this type of autogenic photophore.

While the cytoplasmic organelles that formed in light exposed organs of Pesta suggests that these tissues are photosensitive, there was no indication of pigment migration which was a prominent light-induced change in the abdominal cuticular photophores of *Systellaspis debilis* and *Janicella spinicauda*^23^. Additionally, if microvilli brush borders contain visual opsin proteins as they do in crustacean photoreceptors, then the photoreceptive tissue does not appear to be housed in a separate structure but runs the entire length of each tubule.

As part of this study, we also used RNA-sequencing and phylogenetic methods to help characterize genes known to play a role in light detection. Recent studies using similar methods have shown that cuticular autogenic photophores of oplophorid shrimps (*J. spinicauda* and *S. debilis*) contain visual opsins and other phototransduction genes common to the eye^23,24^. In the present study, we recovered similar light detection genes in the organs of Pesta of the sergestid shrimp *Parasergestes armatus*, though with substantial differences. Our results suggest the organs of Pesta contain most of the major genes involved in the phototransduction pathway and putative long-wavelength sensitive opsins (LWS2), similar to those of other deep-sea shrimp photophores^23,24^. However, a significant difference was the absence of mid-wavelength sensitive opsins (MWS1 and MWS2) in the organs of Pesta, which had been previously recovered in the cuticular photophores of oplophorid shrimps. Due to the number of light organ replicates (n = 4), and identical findings for another sergestid species (DeLeo, unpublished data), we feel confident that the MWS opsins are absent within the organs of Pesta and not missing due to methodological artifact. The significance of MWS absence is unknown, however this may indicate that sergestid and oplophorid shrimps are using light detection differently across the light organ types (internal vs. external). This is not implausible due to the structural and morphological differences that exist across light organs (i.e., organs of Pesta, cuticular photophores and dermal photophores) and the fact that sergestid and oplophorid shrimps are not close relatives across the Decapod Tree of Life^62^. Lastly, although opsin expression does not infer function, future molecular studies that label targeted opsin transcripts and proteins in the photophore are ongoing.

Responses to light were only recorded from the organs of Pesta when the eyes were intact, and experiments in which the electrode was placed in the muscle tissue anterior to the light organ demonstrated that this response to light was not an “echo”, carried through highly conductive (albeit grounded) seawater, of the much larger response occurring in the intact photoreceptors. Latz and Case^5^ demonstrated that restrained live *Sergestes similis* (now known as *Eusergestes similis*) could only be induced to counterilluminate in animals with intact eyes, after exposure to a steady dim background stimulus light for 25 min, while animals exposed to bright ambient room light were no longer able to counterilluminate^21^. The kinetics of the counterillumination responses were consistent with neurally controlled systems, and counterillumination could not be induced in animals in which the eyes had been removed^5^. Latz and Case^5^ suggested that photic induction of bioluminescence involves a blood-born or neurosecretory pathway, a hypothesis that is supported by our data demonstrating no response to light in animals with eyes shielded from the light stimulus. The quick, immediate, albeit small, response to a light stimulus in the organs of Pesta recorded in the current study, with the response latency similar to that recorded directly from the photoreceptors^63^ also supports the Latz and Case hypothesis that there must be direct neural transmission of the response from the eyes to the organs of Pesta, from an as yet unidentified pathway from the photoreceptors to the photophores. Herring^11^ found no morphological evidence of innervation of the organ of Pesta from the eyestalks and attempts to use NeuroVue© to find such a connection in the current study were also unsuccessful, so this remains for future studies. However, the histological data showing changes in organelle structure in the organ of Pesta similar to that seen photoreceptor tissue together with the RNA-seq studies demonstrating that organs of Pesta contain most of the major genes involved in the phototransduction pathway as well as putative long-wavelength sensitive opsins suggests that organs of Pesta themselves can respond to light but possibly at a level that is too small to record with extracellular electrophysiology. We did not stimulate the organs of Pesta with bright white light when the eyes were blocked to see if we could elicit a response, as we were emphasizing utilizing environmentally relevant wavelengths of light; both downwelling light^3^ and the emission spectra from sergestid photophores^12^ peak at blue wavelengths (475 nm).

Earlier studies provided strong evidence that cuticular photophores in two species of oplophorids have the ability to respond to light^23,24^. However, the lack of MWS opsins in the sergestid photophores (organs of Pesta), which were present in the cuticular photophores of these oplophorids^23,24^, suggest that these morphologically different photophores may have different modes of operation. The current study provides two lines of evidence to support the hypothesis that sergestid organs of Pesta are also able to respond to light, but perhaps through a different mechanism: (1) exposure to light resulted in the formation of cytoplasmic organelles identical to those formed during photoreceptor membrane turnover, (2) genes known to play a role in light detection are present. However, the electrophysiological studies indicate that the photophores produce electrophysiological responses to light only when the eyes are intact. Many secretory processes result in the production of the same organelles seen here^64^ and the ultrastructural changes shown here may be due to a stimulus (possibly hormonal) provided by the eyes, as has been shown in sharks^89^. Latz and Case^5^ demonstrated that counterillumination to light stimuli did not occur when the eyes were removed, and the current study demonstrated that no electrophysiological responses are present in the organs of Pesta when light input to the eyes has been blocked.

While this is the first example of light effects on the same organelles found in photophores that are involved in membrane transduction in photoreceptors, there are two examples in other taxa (in addition to the aforementioned study in oplophorids) that support the hypothesis that photoemission and photoreception are functionally coupled. The expression of genes encoding proteins involved in visual transduction, including an opsin that is the same isoform of opsin found in the retina, has been demonstrated in the bacterial light organ of the counterilluminating squid *Euprymna scolopes*^90^. Physiological responses to light were also demonstrated electrophysiologically in this light organ, although it is unclear whether these were isolated light organs, or whether the light stimulus was also seen by the eyes. In this case, the role of light perception has been hypothesized to help the animal host control the population of bioluminescent bacteria to exclude dark mutants that would reduce the light emission to non-functional levels, as well as control the light emission of the organ during counterillumination. An extraocular opsin has also been found in the ventral skin photophores of the lanternshark, with the absorbance spectrum of this extraocular opsin matching the wavelength of light emission in this species^91^. As this species also counterilluminates, and Duchatelet et al.^91^ showed blue light exposure of photophores increased the intracellular concentration of both calcium and IP_3_ (involved in the phototransduction cascade in photoreceptors), they proposed a model of light emission control in this species that involved photoperception of its own luminescence. While putative opsin genes have also been found in the ctenophore *Mnemiopsis leidhyi*^92^ and the burrowing brittle star *Amphiura filiformis*^93^ in their photocytes (light producing cells), there is no evidence that these are involved in control of light emission. Ctenophores do not counterilluminate but use bioluminescence primarily as a defense mechanism, usually invoked by mechanical stimulation^1^ and the same is true for *A. filiformis,* which only luminesces in response to mechanical stimulation^93^. These examples suggest opsins have different functional roles, many of which are yet to be discovered.

The data presented here suggest that suggest that the eyes and organs of Pesta may be communicating to help regulate bioluminescent emissions used during counterillumination. Because the eyes of sergestids cannot detect the bioluminescent emissions from the organs of Pesta, organ photosensitivity might be used to help regulate their own emissions while receiving downwelling light information from the eyes. More studies are needed to (1) determine if the changes in membrane structure and organelles in response to light are due to inherent photosensitivity within the organs or require input from the eyes and (2) verification that there is a neural or hormonal link between the eyes and the organs. Future studies are currently underway using an integrative approach (i.e., behavior and immunohistochemistry) to confirm organ photosensitivity and their potential role in the regulation of counterillumination.

## Methods

Live mesopelagic (200–1000 m) sergestid species with internal organs of Pesta, *Parasergestes armatus* and *Allosergestes sargassi*, were collected aboard the R/V *Walton Smith* in the Florida Straits with a 9 m^2^ Tucker Trawl net and utilized for the histological and genomic studies. Attached to the end of the net was a light-tight, thermally insulated collecting vessel (cod-end) that could be closed at depth^67^ ensuring that animals were not exposed to light and remained at their ambient water temperatures as the net ascended to the surface. Pressure changes are generally not harmful for animals living shallower than 1500 m which do not have air-filled spaces^68^ (swim bladders or air-filled floats), and therefore temperature and light exposure were the major parameters that needed to be controlled. The ambient temperature at their daytime depths was recorded during the trawls with a sensor on the timer used to close the net. Once on shipboard, the cod-end was disconnected from the net and carried into a light-tight room where animals were identified under dim red light, which is virtually invisible to deep-sea species^67^. Samples were either immediately preserved for genomic work or transferred into black, light tight, aerated, 6.5L maintenance containers held inside Koolatrons ©, at their normal daytime ambient temperatures (9–10 °C) to prevent organelle deformities and membrane breakdown that have been shown to occur in mesopelagic crustacean visual systems by warmer temperatures^69^. For the electrophysiological work, *Deosergestes seminudus* was collected on the R/V *Sonne* in the Indian Ocean with a 45 m^2^ opening/closing Tucker Trawl), owned and operated by Professor Justin Marshall, University of Queensland.

### Ultrastructural experiments

Under dim red light, sergestids were placed into sorting trays held inside of Koolatrons. Animals were maintained in complete darkness for 20 min before commencement of experiments^5^. The control group remained in in the dark while the experimental group was exposed to a light level (16 μW cm^−2^ fluorescent bulb room lighting measured with a Gamma Scientific Model S471 optometer with a Nidek 247 Sensor Head) that replicated light levels that caused major changes, including damage, to photoreceptor organelles in crustaceans from low light environments^48,49,69^. Room lighting was used as the light source to avoid cellular damage that may result from heat transfer or UV wavelengths^69^. Following 60-min timed trials, room lighting was turned off and control and experimental animals were transferred under dim red light into a 2% glutaraldehyde 0.05 M sodium cacodylate buffered filtered seawater fixative. Samples spent a minimum of seven days in fixative held at 2 °C in the dark, to allow for better penetration of the fixative and harden tissues for photophore dissection. After fixation, the organs of Pesta were dissected out, washed with 0.05 M sodium cacodylate buffered filtered seawater for three 15-min changes, post-fixed in 1% osmium tetroxide in buffer for 60 min, rinsed in three changes of the buffer, dehydrated in a graded series of ethanol (20%, 50%, 70%), and maintained in 70% ethanol for 48 h. Specimens were further dehydrated in three changes of ethanol (95%, 100%), infiltrated with Spurr embedding resin, and placed into a VWR drying oven at 60 °C for 72 h. Thin sections (90 nm gold) were cut using a Sorvall Porter-Blum MT2-B ultramicrotome fitted with a DKD-diamond knife and transferred to formvar/carbon coated 200 mesh grids. Micrographs were taken at the TEM Core Facility, University of Miami, Miller School of Medicine, using a Phillips CM10 or a Joel 1400 transmission electron microscope.

The formation of cytoplastic organelles that appear to be identical to those formed during photoreceptor membrane turnover (see results), as well as what appears to be “lipid” to organelle contact sites, were identified. “Lipid” diameters in medial and distal tubule regions were measured along their major axis and compared between the controls and light exposed tissues. If a Shapiro–Wilk test determined the data were normally distributed and a Bartlett test showed that variances were homogeneous, statistical analyses were conducted with a one-tailed T-test. If data were not normally distributed and/or if variances were not homogeneous, a non-parametric Mann–Whitney Wilcoxon test was used for statistical analysis. Images were uploaded into the free software ImageJ^70^ and measurements were taken three times and averaged to minimize human error. All statistical analyses were performed in the statistical software R and test statistics were considered significantly different at *p* ≤ 0.05.

### RNA-sequencing

Four biological replicates of *Parasergestes armatus* were immediately preserved in RNAlater and stored at − 80 °C upon live collection. Upon return from the cruise, organs of Pesta were carefully dissected in chilled RNAlater to prevent degradation of nucleic acids and homogenized in TRIzol reagent (ThermoFisher Scientific). Total RNA was extracted following recommendations in DeLeo et al.^71^ and mRNA libraries were prepared using the NEBNext Ultra II Directional RNA Library Prep Kit for Illumina. Pippin Prep (Sage Science) was used for size selection on barcoded libraries and all samples (n = 4) were sequenced on an Illumina HiSeq4000. FastQC^72^ was used to assess quality of raw sequencing data and Trimmomatic v0.36^73^ was used to trim adaptor sequences (parameters: *adapter.clip* 2:30:10:1:true, *crop* 135, *headcrop* 15, *trim.leading* 3, *trim.trailing* 3, *sliding.window* 4:20, *min.read.length* 36). Rcorrector (Song and Florea 2015) and BBnorm were used to error-correct reads and normalize read coverage. Trinity v2.6.5^74^ was used for assembling the tissue-specific transcriptome de novo (minimum contig length of 200 bp, k-mer size of 23) and contamination was removed using Kraken v1.0^75^ with default parameters and NCBI’s (Refseq) bacteria, archaea, and viral databases. BBduk and dedupe (BBTools suite, available at: http://sourceforge.net/projects/bbmap) were used to remove duplicate transcripts and rRNA. Transrate v1.0.3 and BUSCO v3.0.2 (Benchmarking Universal Single-Copy Orthologs^76,77^) were used to assess transcriptome quality and completeness using a reference dataset of orthologous groups (n = 1066) found across Arthropoda^78^. All voucher specimens are ultimately archived in the Florida International Crustacean Collection (FICC, HBG #8487-8490).

The organ of Pesta transcriptome was analyzed using a modified Phylogenetically-Informed Annotation (PIA) Tool^79,80^ which can be used to identify putative visual opsins and phototransduction genes (details outlining these methods^80^. In brief, custom PIA databases include both visual and non-visual opsins as well as other light detection genes, in precomputed phylogenies which enables the discrimination between false positives and/or paralogous genes following a series of BLAST searches. To further characterize putative opsins recovered by PIA, these sequences were aligned with PROMALS3D^81^ using a reference invertebrate-only opsin dataset (n = 283^24,82,83^). This dataset included visual opsins across a range of spectral sensitivities as well as non-visual opsins and related G-protein coupled receptors (GPCR) (see DeLeo and Bracken-Grissom^14^ for a list of all taxa included). Model testing and opsin gene tree reconstruction was done with IQ-TREE^84^ using an LG general amino acid replacement matrix, under a FreeRate model with 9 rate categories, and empirical base frequencies (LG + R9 + F), based on recommendations from ModelFinder^85^. Support was assessed using (1) a Shimodaira–Hasegawa–like approximate likelihood ratio test (SH-aLRT; 10,000 replicates), (2) an approximate Bayes test and (3) an Ultra-fast bootstrap approximation (UFBoot; 10,000 replicates)^86–88^. False positives aligning with non-visual opsins or outgroups were removed.

### Electrophysiological experiments

Experiments were conducted on board the R/V *Sonne* in the Indian Ocean, using a special set-up adapted for shipboard use^67^. Live *Deosergestes seminudus* (the most abundant sergestid in this region) were collected from night trawls, sorted under dim red light, and maintained in the dark at 9–10 °C for 24 h before being used for experiments. Animals used in experiments were attached, under dim red light, to a thin plastic rod with a drop of cyanoacrylic cement, leaving the region with organ of Pesta undisturbed. The rod was suspended in a temperature insulated recording chamber containing seawater maintained at 9–10 °C via chilled antifreeze pumped through cooling coils by a Lauda E1000 circulating water bath. The water bath was on a grounded plate inside a Faraday cage, to eliminate stray electronic noise. The animal was lowered into the water bath until the ventral surface was submerged, leaving the dorsal surface with the organ of Pesta just above the water line. The placement of the animals ensured that their pleopods were able to generate respiratory currents across the gills. Single-ended recordings were made with a metal microelectrode (25–30 mΩ, FHC, Inc.), inserted thru the carapace on various regions of the photophore. The response was amplified with an FHC model XCell-3 microelectrode utilizing a high impedance probe to eliminate electrode polarization artifacts. The seawater bath was grounded with an AgCl-coated wire. Light from 175 W xenon lamp, filtered to 490 nm with a CM110 monochromator (Spectral Products), was transmitted to the photophore through a small hole in the cage via a randomized fiber light guide (EXFO). This narrow band blue light was used to simulate the wavelength of downwelling light at normal daytime depths (400–600 M) of this species, as this is the only light available for photophores to utilize if they are indeed light sensitive. A shutter (Uniblitz Model VS25) and neutral density filter wheel, under computer control using a custom program written in LabView (National Instruments), provided light stimuli of varying durations and irradiances. The highest irradiance used was 1 × 10^14^ photons/cm^2^/s^1^. Various durations of light stimuli were tested (from 100 ms to 1 s).

The AC recordings were digitized and stored using a custom program written in LabView (National Instruments). In the initial experiments, the electrode was placed subcorneally in the eye, to verify the efficacy of the light shield preventing light directed onto the organ of Pesta from being detected by the eyes. Subsequent experiments involved recording responses, or lack thereof, with the electrode in (1) the anterolateral organ with shielded eyes, (2) anterolateral organ with unshielded eyes, and (3) the muscle tissue anterior to the organ of Pesta. Digitized data were downloaded from LabView to Excel to make the graphs in Fig. 8.

## Data Availability

The RNAseq data for the organs of Pesta are publicly available on the NCBI’s Sequence Read Archive (SRA) database under BioProject: PRJNA690607. The assembled transcriptome, putative opsins and alignments are open-access via Dryad (10.5061/dryad.9ghx3ffp8). This is contribution #X from the Coastlines and Oceans Division in the Institute of Environment at Florida International University.
